# How to keep a patient support group running?

**DOI:** 10.1186/1746-160X-8-S1-I13

**Published:** 2012-05-25

**Authors:** Karla Padilla Gomez, Elzbieta Blum

**Affiliations:** 1 Asociación Mexicana de Displasia Ectodérmica, Toluca, Mexico; 2 Ectodermal dysplasia patient support group, Poznan, Poland

## 

The achievements and challenges of modern society have an important impact on patients with rare diseases and on the patient support groups that have been established in many countries. Although people are now able to exchange information from one corner of the world to another just in a minute via internet, personal relationships and wholehearted personal communication have remained the best ways to share experiences and to support each other. Nowadays patient organizations may also contribute to shaping patient-centred health policies by describing their experiences and expectations on diagnosis and care. Networking with medical experts often leads to improved access to medical services and novel research findings. We will report on the experiences of two young patient associations in Mexico and Poland.

